# Systemic Immune Signatures of Endoscopic–Histologic Discordance in Inflammatory Bowel Disease: A Pilot Study

**DOI:** 10.3390/jcm15093319

**Published:** 2026-04-27

**Authors:** Nikolaos Martinos, Christos Kroupis, Maria Gypari, Georgios Kranidiotis, Christos Karakoidas, Marina Konstantinou, Andreas C. Lazaris, Georgia-Eleni Thomopoulou

**Affiliations:** 1 Gastroenterology Department, Naval Hospital of Athens, 70 Dinokratous St., 115 21 Athens, Greece; g.kranidiotis@gmail.com (G.K.); chkarako@gmail.com (C.K.); 2 Department of Clinical Biochemistry, Attikon University General Hospital, National and Kapodistrian University of Athens, 124 62 Athens, Greece; ckroupis@med.uoa.gr; 3 Department of Pathology, Naval Hospital of Athens, 70 Dinokratous St., 115 21 Athens, Greece; mgypari1505@gmail.com (M.G.); marinakonst@yahoo.gr (M.K.); 4 First Department of Pathology, School of Medicine, National and Kapodistrian University of Athens, 115 27 Athens, Greece; alazaris@med.uoa.gr; 5 Cytopathology Department, Attikon University General Hospital, School of Medicine, National and Kapodistrian University of Athens, 124 62 Athens, Greece; gthomop@med.uoa.gr

**Keywords:** inflammatory bowel disease, ulcerative colitis, Crohn’s disease, Geboes score, interleukin-10, interleukin-23, cytokine signaling, histologic remission, biomarkers

## Abstract

**Background:** Endoscopic remission is a central therapeutic target in inflammatory bowel disease (IBD); however, histologic inflammation may persist despite apparent mucosal healing. The biological mechanisms underlying endoscopic–histologic discordance remain incompletely defined. We aimed to characterize systemic cytokine patterns associated with discordant disease. **Methods:** In this prospective cross-sectional study, 59 patients with IBD undergoing clinically indicated colonoscopy underwent concurrent endoscopic and histologic assessment. Endoscopic remission was defined as a Mayo endoscopic subscore ≤ 1 for ulcerative colitis and SES-CD ≤ 2 for Crohn’s disease. Histologic healing was defined as a Geboes score < 2.0. Patients were classified into concordant remission, discordant disease (endoscopic remission with persistent histologic activity), and concordant active disease. Circulating interleukin (IL)-10, IL-23, and C-reactive protein (CRP) levels were compared across phenotypes using nonparametric methods. Associations with discordant disease were evaluated using Firth penalized logistic regression, and model discrimination was assessed using receiver operating characteristic (ROC) analysis with bootstrap internal validation. **Results:** Among 36 patients in endoscopic remission, 14 (38.9%) exhibited persistent histologic activity. Discordant patients demonstrated significantly lower IL-10 and higher IL-23 concentrations compared with concordant remission (both *p* < 0.001), whereas CRP did not differ significantly. Across phenotypes, IL-10 decreased progressively, while IL-23 increased stepwise (both *p* < 0.001). In multivariable analysis, lower IL-10 (OR 0.0014; 95% CI 0.000003–0.576; *p* = 0.032) and higher IL-23 (OR 16.94; 95% CI 1.90–151.32; *p* = 0.011) were independently associated with discordant disease. A model incorporating IL-10 and CRP demonstrated strong discrimination (AUC 0.925), with further improvement after inclusion of IL-23 (AUC 0.994), although these estimates should be interpreted with caution given the limited sample size. **Conclusions:** Endoscopic–histologic discordance is associated with a distinct systemic cytokine profile characterized by reduced IL-10 and elevated IL-23 levels, despite low CRP concentrations. These findings suggest incomplete restoration of immune regulatory balance and highlight the potential role of circulating cytokines, particularly IL-10, in identifying ongoing microscopic inflammation. These results are exploratory and hypothesis-generating and require validation in larger, prospective cohorts before clinical application.

## 1. Introduction

Inflammatory bowel disease (IBD), encompassing ulcerative colitis and Crohn’s disease, is characterized by chronic relapsing intestinal inflammation driven by dysregulated immune responses [1]. Over the past decade, therapeutic strategies have shifted from symptom-based management toward objective treatment targets within a treat-to-target framework. Endoscopic remission has emerged as a key therapeutic goal and is associated with improved long-term outcomes.

However, increasing evidence suggests that endoscopic remission does not invariably reflect complete resolution of mucosal inflammation [2,3]. Histologic activity may persist despite apparent macroscopic healing and has been associated with increased risk of relapse, corticosteroid use, and disease progression. This phenomenon of endoscopic–histologic discordance underscores the complexity of mucosal immune regulation and raises important questions regarding the biological underpinnings of residual microscopic inflammation.

While the clinical implications of persistent histologic activity have been increasingly recognized, the systemic immune profile of patients with discordant disease remains incompletely defined. Conventional inflammatory markers such as C-reactive protein (CRP) provide indirect estimates of systemic inflammation but often fail to discriminate subtle differences in microscopic disease activity, particularly in patients who appear endoscopically quiescent [4,5,6,7,8]. A more detailed characterization of systemic regulatory and effector immune patterns may offer insights into the persistence of histologic inflammation despite endoscopic improvement.

Interleukin-10 (IL-10) is a central immunoregulatory cytokine that plays a pivotal role in maintaining intestinal immune homeostasis by suppressing antigen-presenting cell activation and limiting pro-inflammatory cytokine production. Conversely, interleukin-23 (IL-23) is a key driver of pathogenic inflammatory pathways and promotes sustained effector T-cell responses. These cytokines represent complementary axes of immune regulation and amplification that may influence the balance between mucosal healing and persistent inflammation.

Although prior studies have examined cytokine expression in relation to clinical or endoscopic activity, limited data are available regarding systemic immune signatures specifically associated with endoscopic–histologic discordance. Understanding whether patients with persistent microscopic inflammation exhibit distinct circulating cytokine patterns may provide biological context for discordant disease states and help refine disease monitoring strategies. Within modern treat-to-target strategies, defining the biological correlates of incomplete remission has become increasingly important for optimizing disease monitoring and therapeutic decision-making [9,10,11,12].

In this prospective cross-sectional study, we aimed to characterize systemic inflammatory and regulatory profiles across predefined endoscopic–histologic phenotypes in IBD [13,14]. Specifically, we investigated whether patients with endoscopic remission but persistent histologic activity display distinct circulating cytokine patterns compared with those achieving concordant remission or concordant active disease [15,16,17,18,19].

## 2. Materials and Methods

### 2.1. Study Design and Participants

This analysis was conducted within a prospectively established, single-center observational cohort at the Hepato-Gastroenterology Unit of the Naval Hospital of Athens, Greece.

This study was conducted within a prospectively established observational cohort, with predefined data collection prior to outcome assessment.

The parent cohort included consecutive adult patients (≥18 years) with an established diagnosis of inflammatory bowel disease (IBD), comprising ulcerative colitis (UC) and Crohn’s disease (CD), who underwent clinically indicated colonoscopy between March 2025 and August 2025. Patient screening and eligibility assessment commenced in March 2025; however, all prospective enrollment and data collection were performed only after ethics approval was obtained in April 2025.

While the broader study framework was designed to investigate circulating cytokines in relation to disease activity, the present analysis was predefined to examine systemic immune patterns across endoscopic–histologic phenotypes, with a particular focus on discordant disease within endoscopic remission. The present analysis is cross-sectional in nature, reflecting a single time-point evaluation within the prospective cohort. This study is not retrospective; it represents a cross-sectional analysis within a prospectively established cohort with predefined data collection.

The diagnosis of IBD was established according to standard clinical, endoscopic, radiologic, and histopathologic criteria in accordance with international consensus guidelines. Eligible patients included those undergoing colonoscopy for routine disease assessment or clinical evaluation and receiving standard IBD therapies, including biologic agents, immunomodulators, or aminosalicylates.

Exclusion criteria were predefined and included age < 18 years, pregnancy, active infection at the time of enrollment, known hereditary disease, history of or active malignancy, severe organ failure, and antibiotic use within four weeks prior to study inclusion.

Given the specific focus on cytokine-based immune profiling and the inclusion of interleukin-23 (IL-23) as a key analyte, patients receiving monoclonal antibodies targeting the IL-23 pathway, or with exposure within the preceding 12 months, were excluded in order to minimize pharmacologic interference with circulating cytokine measurements. This restriction was applied to preserve the biological interpretability of systemic cytokine measurements in the context of immune phenotype characterization.

Patients with incomplete endoscopic, histologic, or cytokine data were additionally excluded from the analysis.

The present study focused exclusively on patients with complete IBD data (*n* = 59). Healthy controls enrolled in the broader cohort were not included, as the objective of this analysis was to characterize immune profiles within clinically relevant endoscopic–histologic phenotypes rather than to perform case–control comparisons. Given the exploratory and hypothesis-generating nature of the study, and the absence of prior data to inform effect size estimation for systemic cytokine patterns in endoscopic–histologic discordance, a formal sample size calculation was not performed.

### 2.2. Ethical Approval

The study was conducted in accordance with the Declaration of Helsinki (1975, revised in 2013). The protocol was approved by the Institutional Review Board of the Naval Hospital of Athens (protocol code 3366, approved on 11 April 2025) and by the Institutional Review Board of Attikon University Hospital (protocol code 219, approved on 8 April 2025). Written informed consent was obtained from all participants prior to enrollment.

### 2.3. Endoscopic and Histologic Assessment

Colonoscopy was performed according to standard clinical practice. Endoscopic activity in ulcerative colitis (UC) was assessed using the Mayo endoscopic subscore, and endoscopic remission was defined a priori as a Mayo score ≤ 1. In Crohn’s disease (CD), endoscopic activity was evaluated using the Simple Endoscopic Score for Crohn’s Disease (SES-CD), and endoscopic remission was defined as SES-CD ≤ 2.

Mucosal biopsy specimens were obtained during colonoscopy from intestinal segments according to standard clinical practice. Biopsies were systematically collected from areas of both macroscopically normal and endoscopically inflamed mucosa, when present, in order to enable comprehensive assessment of microscopic inflammatory activity across different mucosal compartments.

All biopsy specimens were immediately fixed in formalin and processed according to standard histopathologic protocols. Histologic activity was independently evaluated by experienced gastrointestinal pathologists who were blinded to clinical, endoscopic, and laboratory data, and graded using the Geboes scoring system.

Histologic healing was defined as a Geboes score < 2.0. For the purposes of phenotype classification, the highest Geboes score per patient was used, reflecting the maximal degree of microscopic inflammatory activity and enabling alignment with clinically relevant endoscopic–histologic phenotypes.

### 2.4. Definition of Endoscopic–Histologic Phenotypes

Patients were categorized into three predefined endoscopic–histologic phenotypes based on the combination of endoscopic and histologic findings. This classification framework was selected to capture clinically relevant concordance and discordance between macroscopic and microscopic disease activity.

Concordant remission was defined as the coexistence of endoscopic remission and histologic healing. Discordant disease was defined as endoscopic remission accompanied by persistent histologic inflammatory activity. Concordant active disease was defined as the presence of both endoscopic and histologic activity.

Two patients with endoscopic activity but histologic healing were identified. Given the small number of such cases and the potential for unstable estimates, these patients were excluded from three-group comparative analyses but were retained in descriptive summaries.

The primary analysis was prespecified and restricted to patients in endoscopic remission, comparing concordant remission with discordant disease. This approach was chosen to specifically evaluate systemic immune patterns associated with residual histologic activity in the setting of apparent mucosal healing, a clinically relevant scenario within contemporary treat-to-target strategies.

### 2.5. Blood Sampling and Cytokine Quantification

Peripheral venous blood samples were obtained immediately prior to colonoscopy and before any endoscopic manipulation. Serum was separated by centrifugation within two hours of collection and stored at −80 °C until analysis.

Samples were stored for comparable durations across study groups. The first collected sample was analyzed approximately six months after collection, and the overall median storage duration was approximately three months. All samples were analyzed in batch during the same study period to minimize inter-assay variability. Each sample underwent a single freeze–thaw cycle prior to cytokine quantification. Pre-analytical handling procedures were identical across study groups to reduce the risk of differential degradation bias. To ensure assay reproducibility, all cytokine measurements were performed in duplicate, and mean values were used for analysis. All samples were analyzed within the same assay batch to minimize inter-assay variability. According to the manufacturer’s specifications, the intra-assay and inter-assay coefficients of variation were <10% for both IL-10 and IL-23. Standard calibration curves and internal quality controls were applied in accordance with the manufacturer’s protocol. All laboratory procedures were conducted under standardized conditions by experienced personnel blinded to clinical data.

### 2.6. IL-10 Quantification

Serum interleukin-10 (IL-10) concentrations were measured using a commercially available sandwich enzyme-linked immunosorbent assay (Human IL-10 Quantikine ELISA Kit, D1000B; R&D Systems, Minneapolis, MN, USA) according to the manufacturer’s instructions. All samples and standards were measured in duplicate, and mean values were used for statistical analysis. Optical density was measured at 450 nm with wavelength correction using a Bio-Tek ELx800 microplate reader (BioTek Instruments, Winooski, VT, USA).

The manufacturer-reported lower limit of detection (LOD) was 3.9 pg/mL. The assay calibration curve provided quantitative estimates across the full fitted range, including concentrations below the nominal detection threshold. Accordingly, IL-10 concentrations were retained as assay-reported values for continuous analyses in order to preserve the underlying data distribution and avoid artificial truncation.

For descriptive tabular summaries, values below the detection limit were imputed as half the detection threshold (LOD/2) to prevent zero-inflated distributions. In addition, IL-10 detectability (≥3.9 pg/mL) was explored in secondary categorical analyses.

### 2.7. IL-23 Quantification

Serum interleukin-23 (IL-23) concentrations were measured using a commercially available sandwich enzyme-linked immunosorbent assay (Human IL-23 Quantikine ELISA Kit, D2300B; R&D Systems, Minneapolis, MN, USA) according to the manufacturer’s instructions. All samples were measured in duplicate, and mean values were used for analysis.

The manufacturer-reported lower limit of detection (LOD) was 16.3 pg/mL. As with IL-10, the assay calibration curve enabled quantitative estimation across the full fitted range, including values below the nominal detection threshold. Therefore, IL-23 concentrations were analyzed as continuous variables using the assay-reported values.

For descriptive purposes, values below the detection limit were imputed as half the detection threshold (LOD/2). IL-23 detectability (≥16.3 pg/mL) was additionally evaluated in categorical analyses.

The combined assessment of IL-10 and IL-23 was intended to provide a simplified representation of regulatory and pro-inflammatory immune signaling axes within the context of endoscopic–histologic phenotypes.

### 2.8. Handling of Detection Limits

Cytokine concentrations were analyzed primarily as continuous variables using the assay-reported values, including measurements below the nominal limits of detection (LOD), in order to preserve the underlying distribution of the data and avoid artificial truncation.

The manufacturer-reported LODs were 3.9 pg/mL for interleukin-10 (IL-10) and 16.3 pg/mL for interleukin-23 (IL-23). Although these thresholds define the lower limits of reliable detection, the assay calibration curves provided quantitative estimates across the full fitted range, including low-concentration values below the nominal LOD.

For descriptive tabular summaries, values below the detection limit were imputed as half the respective detection threshold (LOD/2) to avoid zero-inflated distributions. However, no imputation was applied in primary continuous analyses.

In addition, cytokine detectability (≥3.9 pg/mL for IL-10 and ≥16.3 pg/mL for IL-23) was explored in secondary categorical analyses. These analyses were considered supportive and did not replace the primary continuous-variable approach.

### 2.9. Laboratory Measurements

Routine laboratory parameters, including C-reactive protein (CRP), hemoglobin, and white blood cell (WBC) count, were measured using standardized automated assays in the hospital’s accredited clinical laboratory, in accordance with routine clinical practice.

These parameters were included to provide complementary information on systemic inflammatory status and overall hematologic profile. CRP was specifically incorporated in sensitivity analyses as a marker of systemic inflammation, whereas hemoglobin and WBC counts were used for descriptive characterization of the study population across endoscopic–histologic phenotypes.

### 2.10. Treatment Classification

At the time of colonoscopy, patients were categorized according to ongoing maintenance therapy, including no treatment, 5-aminosalicylic acid (5-ASA), biologic therapy, or combination therapy.

Given the known immunomodulatory effects of therapeutic agents on cytokine profiles, treatment exposure was considered a potential source of confounding. However, due to the modest sample size and the phenotype-driven focus of the analysis, treatment was not included in the primary multivariable model in order to preserve model stability.

Treatment distribution was therefore primarily considered in descriptive analyses and interpreted in the context of disease subtype and clinical phenotype.

### 2.11. Study Outcomes

The primary outcome of the study was the identification of systemic cytokine patterns associated with discordant disease among patients in endoscopic remission, defined as the presence of endoscopic remission with persistent histologic inflammatory activity.

This outcome was selected to specifically capture residual microscopic inflammation in the setting of apparent mucosal healing, a clinically relevant form of endoscopic–histologic discordance within contemporary treat-to-target strategies.

Secondary analyses evaluated differences in systemic inflammatory markers across three predefined endoscopic–histologic phenotypes (concordant remission, discordant disease, and concordant active disease), in order to characterize broader immune profiles across distinct disease states.

Additional exploratory analyses assessed independent associations between circulating cytokine concentrations and discordant status using penalized multivariable regression models. These analyses were considered hypothesis-generating and were not intended to establish predictive performance.

### 2.12. Statistical Analysis

Continuous variables are presented as median with interquartile range (IQR), and categorical variables as counts and percentages. Given the right-skewed distribution of several continuous variables, nonparametric statistical methods were applied throughout the analyses. All statistical tests were two-sided, and a *p* value < 0.05 was considered statistically significant.

For the primary analysis restricted to patients in endoscopic remission, comparisons between concordant remission and discordant disease were performed using the Mann–Whitney U test for continuous variables and Fisher’s exact test for categorical variables.

For comparisons across the three predefined endoscopic–histologic phenotypes (concordant remission, discordant disease, and concordant active disease), continuous variables were analyzed using the Kruskal–Wallis test. When overall significance was observed, post hoc pairwise comparisons were conducted using the Mann–Whitney U test with Holm adjustment for multiple testing.

Continuous cytokine analyses were performed using assay-reported concentrations in order to preserve the underlying distribution of the data. Additional categorical analyses explored cytokine detectability according to assay-specific thresholds (≥3.9 pg/mL for IL-10 and ≥16.3 pg/mL for IL-23).

Given the phenotype-driven and exploratory nature of the analysis, and the limited number of discordant cases, multivariable modeling was intentionally restricted to preserve interpretability and reduce the risk of overfitting. Discordant status among patients in endoscopic remission was evaluated using Firth penalized logistic regression.

The primary multivariable model included z-standardized, log-transformed IL-10 and IL-23 concentrations to account for differences in scale and distribution. A sensitivity model additionally adjusted for C-reactive protein (CRP) and sex as potential confounders. Odds ratios (ORs) and corresponding 95% confidence intervals (CIs) were derived from penalized model estimates.

These multivariable analyses were considered exploratory and hypothesis-generating and were not intended to establish predictive performance.

All statistical analyses were performed using R software (version 4.3.1; R Foundation for Statistical Computing, Vienna, Austria).

## 3. Results

### 3.1. Study Population and Phenotype Classification

A total of 59 patients with inflammatory bowel disease (IBD) underwent concurrent endoscopic and histologic evaluation and were included in the study cohort.

Endoscopic remission was observed in 36 patients (61.0%), whereas 23 patients (39.0%) demonstrated endoscopic activity. Histologic healing was present in 24 patients (40.7%), while 35 patients (59.3%) had persistent histologic activity.

Among patients in endoscopic remission (*n* = 36), 22 (61.1%) achieved concordant remission, while 14 (38.9%) exhibited persistent histologic inflammation, corresponding to the discordant phenotype.

Among patients with endoscopic activity (*n* = 23), 21 demonstrated concordant histologic activity, whereas 2 patients showed histologic healing. Due to the small number of such cases, these patients were excluded from phenotype-based comparative analyses but were retained in descriptive summaries.

**Figure 1:**
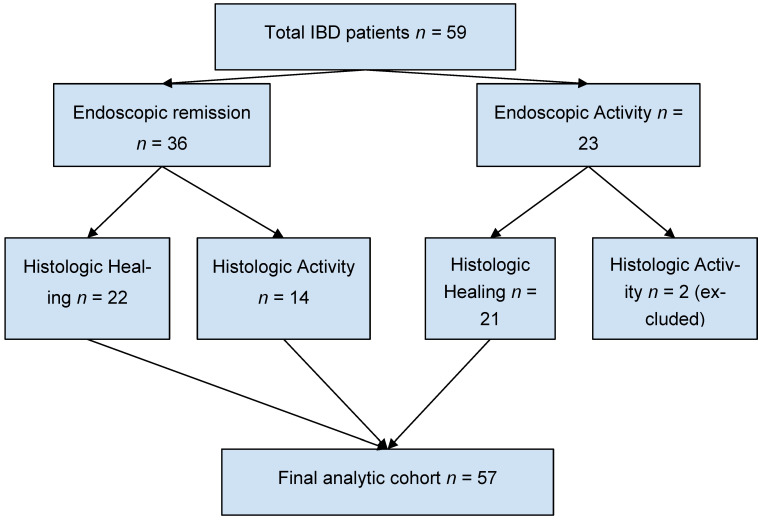
Study flow diagram. Flow diagram of patient inclusion and phenotype classification. A total of 59 patients with inflammatory bowel disease underwent concurrent endoscopic and histologic evaluation. Patients were categorized into concordant remission, discordant disease, and concordant active disease. Two patients with endoscopic activity and histologic healing were excluded from phenotype-based comparative analyses.

The final analytic cohort for phenotype-based comparisons therefore consisted of 57 patients (Figure 1).

### 3.2. Baseline Characteristics According to Endoscopic–Histologic Phenotype

Baseline characteristics stratified according to endoscopic–histologic phenotype are presented in Table 1. Detailed Montreal classification data are presented in Supplementary Table S1.

In contrast, significant differences were observed in systemic inflammatory markers. C-reactive protein (CRP) levels were lowest in patients with concordant remission and highest in those with concordant active disease (*p* = 0.002). Similarly, interleukin-10 (IL-10) concentrations were highest in concordant remission and progressively decreased across discordant and concordant active phenotypes (*p* < 0.001), whereas interleukin-23 (IL-23) levels demonstrated the opposite pattern, with higher concentrations observed in discordant and concordant active disease (*p* < 0.001).

**Figure 2:**
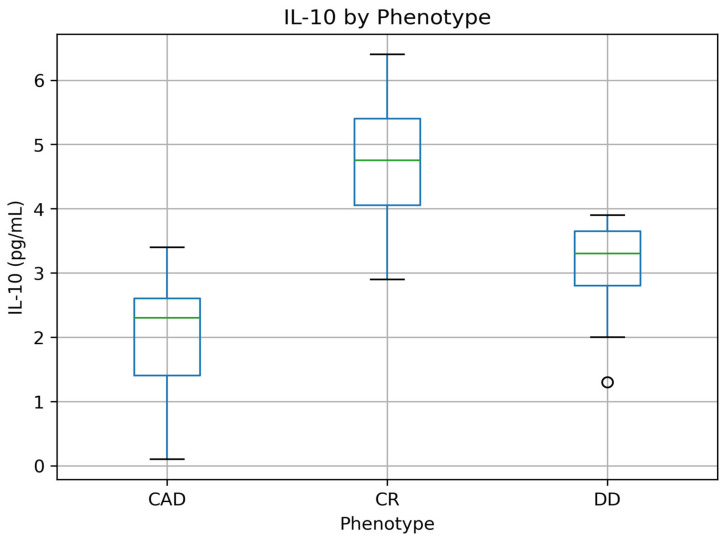
Serum interleukin-10 (IL-10) concentrations across concordant remission (CR), discordant disease (DD), and concordant active disease (CAD). Values are presented as median and interquartile range. The central line represents the median, the box indicates the interquartile range (IQR), and the whiskers represent the minimum and maximum values. Differences between groups were assessed using the Kruskal–Wallis test.

**Figure 3:**
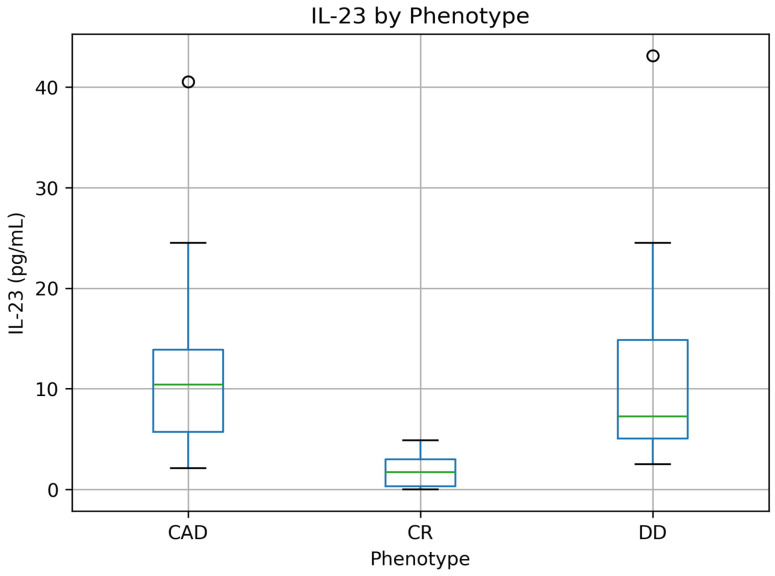
Serum interleukin-23 (IL-23) concentrations across concordant remission (CR), discordant disease (DD), and concordant active disease (CAD). Values are presented as median and interquartile range. The central line represents the median, the box indicates the interquartile range (IQR), and the whiskers represent the minimum and maximum values. Differences between groups were assessed using the Kruskal–Wallis test.

The distribution of circulating cytokine concentrations across endoscopic–histologic phenotypes is illustrated in Figure 2 and Figure 3. Interleukin-10 (IL-10) levels were highest in patients with concordant remission and progressively decreased across discordant and concordant active disease, whereas interleukin-23 (IL-23) demonstrated the opposite pattern, with higher concentrations observed in discordant and concordant active phenotypes.

### 3.3. Endoscopic–Histologic Relationship

The relationship between endoscopic and histologic findings is presented in Table 2.

Overall, histologic–endoscopic discordance was observed in 16 of 59 patients (27.1%). Among patients in endoscopic remission (*n* = 36), 14 (38.9%) demonstrated persistent histologic activity, corresponding to the discordant phenotype.

Conversely, among patients with endoscopic activity (*n* = 23), histologic healing was observed in 2 patients (8.7%). There was a statistically significant association between endoscopic and histologic status (Pearson χ^2^ test, *p* < 0.001), although substantial discordance remained evident, particularly among patients classified as being in endoscopic remission.

### 3.4. Association Between Circulating Cytokines and Discordant Disease

To evaluate whether circulating cytokine levels were associated with histologic activity among patients in endoscopic remission, analyses were restricted to patients in endoscopic remission (*n* = 36).

Within this subgroup, patients with discordant disease (*n* = 14) demonstrated significantly lower interleukin-10 (IL-10) concentrations compared with those with concordant remission (*n* = 22) (*p* < 0.001). In contrast, interleukin-23 (IL-23) levels were significantly higher in patients with discordant disease (*p* < 0.001).

The results of regression analyses are presented in Table 3.

To further assess independent associations, discordant status was modeled using Firth penalized logistic regression. In the primary multivariable model including z-standardized log-transformed IL-10 and IL-23 concentrations, lower IL-10 and higher IL-23 levels were independently associated with discordant disease.

In a sensitivity model additionally adjusting for C-reactive protein (CRP) and sex, the direction of these associations remained unchanged.

Overall, these findings indicate that discordant disease is characterized by a distinct systemic cytokine profile, even in the absence of endoscopic activity.

### 3.5. IL-10 Detectability and Cytokine Patterns in Endoscopic Remission

To further explore the clinical relevance of circulating cytokines, an exploratory analysis was performed using IL-10 detectability (≥3.9 pg/mL) as a binary marker among patients in endoscopic remission.

Non-detectable IL-10 levels were more frequently observed in patients with discordant disease compared with those in concordant remission (*p* < 0.001).

The diagnostic performance of IL-10 detectability for identifying histologic activity is presented in Table 4. Non-detectable IL-10 demonstrated high sensitivity and negative predictive value for histologic activity, indicating that detectable IL-10 levels were strongly associated with histologic healing.

Consistent with these findings, interleukin-23 (IL-23) concentrations remained higher in patients with discordant disease in exploratory analyses; however, no threshold-based analysis was performed for IL-23 due to the absence of a predefined clinically meaningful cutoff.

These findings are consistent with the primary continuous-variable analyses and support the complementary roles of IL-10 and IL-23 in characterizing discordant disease.

### 3.6. Discriminatory Performance

Exploratory assessment of model discrimination was performed for the identification of discordant disease among patients in endoscopic remission. The results are presented in Table 5.

**Figure 4:**
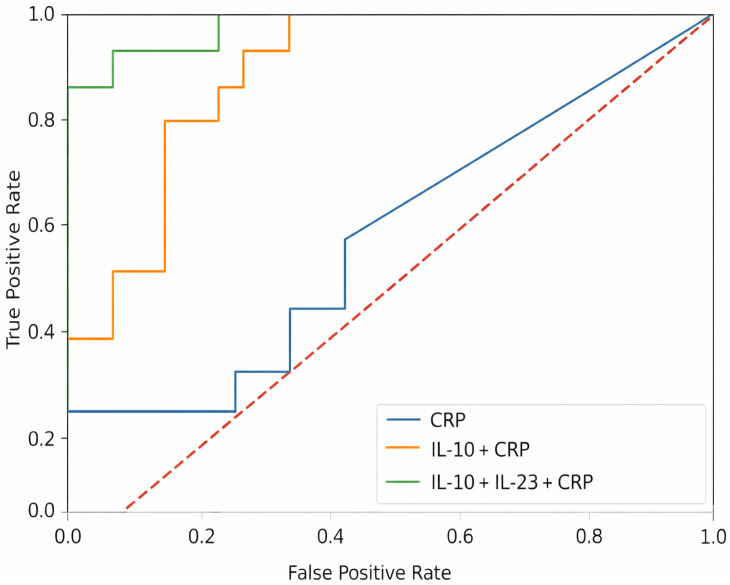
Receiver operating characteristic (ROC) curves for models predicting discordant disease among patients in endoscopic remission. ROC curves are shown for the base model (CRP), the reduced model (IL-10 + CRP), and the multivariable model (IL-10 + IL-23 + CRP). The addition of IL-10 substantially improved model discrimination, while IL-23 provided a modest incremental contribution. The diagonal dashed line represents chance discrimination.

A base model including C-reactive protein (CRP) demonstrated modest discriminatory performance. The addition of interleukin-10 (IL-10) substantially improved model discrimination, while further inclusion of interleukin-23 (IL-23) provided only a modest incremental improvement. The corresponding receiver operating characteristic (ROC) curves are presented in Figure 4.

Internal validation using bootstrap resampling suggested stable model performance, with no evidence of substantial optimism. Calibration assessment indicated acceptable agreement between predicted and observed probabilities, although these estimates should be interpreted with caution given the modest sample size.

These findings suggest that IL-10 contributes the majority of the discriminatory signal, whereas IL-23 provides complementary, but less pronounced, additional information. However, these analyses were exploratory and should be interpreted with caution.

## 4. Discussion

In this prospective observational study, circulating cytokine profiles were associated with histologic–endoscopic discordance among patients with inflammatory bowel disease (IBD) in endoscopic remission [20,21]. Specifically, lower interleukin-10 (IL-10) and higher interleukin-23 (IL-23) concentrations were consistently observed in patients with persistent histologic activity despite apparent mucosal healing. These associations remained robust after multivariable adjustment, suggesting that systemic immune signatures may capture dimensions of disease activity not reflected by endoscopic assessment alone.

Histologic–endoscopic discordance represents a clinically relevant and increasingly recognized phenomenon [22]. In the present cohort, nearly 40% of patients in endoscopic remission exhibited ongoing microscopic inflammation, reinforcing prior observations that endoscopic healing does not equate to complete mucosal immune resolution. These findings underscore the conceptual limitation of endoscopy as a surrogate endpoint and support the notion that deeper layers of disease activity persist beyond macroscopic normalization.

The observed cytokine patterns provide a biologically coherent framework for this discordance. IL-10 is a central regulatory cytokine with a well-established role in maintaining intestinal immune homeostasis. Loss-of-function mutations in IL-10 signaling pathways are causally linked to severe colitis, highlighting its fundamental importance. However, the interpretation of circulating IL-10 levels remains context-dependent. IL-10 is inducible in response to inflammatory stimuli and may be elevated during active disease as part of a compensatory regulatory response [23,24,25,26,27,28]. Therefore, systemic IL-10 concentrations do not directly reflect disease quiescence, but rather the balance between inflammatory and counter-regulatory pathways.

Within this framework, the association between higher IL-10 levels and histologic healing observed in the present study may reflect a state in which regulatory pathways are sufficiently dominant to suppress ongoing mucosal inflammation [29,30,31,32]. This interpretation aligns with emerging concepts of immune restoration, in which the resolution of inflammation is characterized not only by the attenuation of pro-inflammatory signals but also by the re-establishment of regulatory control [33,34,35,36].

In contrast, IL-23 is a key pro-inflammatory cytokine involved in the maintenance of Th17-mediated immune responses and has been strongly implicated in IBD pathogenesis [34,35,36,37]. The elevated IL-23 levels observed in patients with discordant disease support the presence of persistent immune activation despite endoscopic remission [38,39,40,41,42]. Notably, IL-23 concentrations in discordant patients were comparable to those observed in concordant active disease, suggesting that IL-23 may more closely reflect underlying immunologic activity than endoscopic status. Taken together, the combined pattern of reduced IL-10 and elevated IL-23 is consistent with a shift toward a pro-inflammatory immune milieu and provides a mechanistic explanation for persistent microscopic inflammation [42,43,44,45,46].

Importantly, these findings should be interpreted within the context of an exploratory, hypothesis-generating study design. The analyses were not intended to establish clinical thresholds or definitive biomarker performance, and causal inferences cannot be drawn. The observed associations instead provide signal detection that may inform future mechanistic and translational research.

The discriminatory performance analyses further support the potential relevance of these cytokines. The addition of IL-10 to conventional inflammatory markers substantially improved model discrimination, while IL-23 contributed additional, albeit more modest, incremental value. However, the very high AUC observed in the multivariable model warrants careful interpretation. In the context of a relatively small sample size, such performance metrics may reflect overfitting despite the use of penalized regression and bootstrap internal validation. Accordingly, these results should be viewed as indicative of potential signal rather than definitive evidence of predictive utility.

From a clinical perspective, IL-10 may represent a complementary biomarker reflecting regulatory immune activity, whereas IL-23 may capture ongoing pro-inflammatory signaling. This conceptual distinction highlights the potential value of integrating markers of both inflammatory and regulatory pathways, rather than relying solely on traditional inflammatory indices such as C-reactive protein (CRP). The absence of significant differences in CRP across discordant and concordant remission groups further emphasizes the limitation of conventional systemic markers in detecting microscopic disease activity.

Several limitations merit consideration. The study was conducted at a single tertiary center and included a modest number of patients, particularly within subgroup analyses, which may limit generalizability and contribute to statistical imprecision. In addition, no formal sample size calculation was performed, as the study was designed as an exploratory, hypothesis-generating analysis in the absence of prior data to inform effect size estimation for systemic cytokine patterns in endoscopic–histologic discordance. Accordingly, the present findings should be interpreted with appropriate caution and considered hypothesis-generating rather than definitive. Although Firth penalized regression was used to mitigate small-sample bias and reduce the risk of quasi-separation, the resulting effect estimates remain imprecise, as reflected by wide confidence intervals. Accordingly, the present findings should be interpreted with appropriate caution and considered hypothesis-generating rather than definitive. Although Firth penalized regression was used to mitigate small-sample bias and reduce the risk of quasi-separation, the resulting effect estimates remain imprecise, as reflected by wide confidence intervals. In addition, the very high AUC observed in the multivariable model may reflect model overfitting in the context of limited sample size and should not be interpreted as evidence of definitive predictive performance. Despite the use of penalized methods and internal validation, the risk of residual overfitting cannot be fully excluded. The cross-sectional design precludes assessment of temporal dynamics and does not allow evaluation of longitudinal outcomes, including relapse, treatment response, or disease progression. Although the data were derived from a prospectively established cohort, the present analysis reflects a single time-point evaluation and should not be interpreted as a longitudinal or causal assessment. Accordingly, the ability of these cytokines to predict future clinical trajectories remains uncertain. The pooling of ulcerative colitis and Crohn’s disease in the primary analysis represents a pragmatic approach to preserve statistical power and reflect clinical heterogeneity; however, it does not imply biological equivalence between disease subtypes. The absence of statistically significant interaction should not be interpreted as evidence of homogeneity, and disease-specific validation is required. Future studies with adequately powered disease-specific cohorts are warranted to confirm the observed associations. Treatment exposure represents an additional potential source of confounding, given the known immunomodulatory effects of biologic therapies on cytokine profiles and the imbalance in treatment distribution across subgroups. Although treatment was incorporated into multivariable models using a simplified classification, more granular characterization of therapeutic exposure was not feasible, and residual confounding cannot be excluded. Therefore, the independent contribution of cytokine levels should be interpreted within the context of potential treatment-related effects. Fecal calprotectin, a widely used and validated marker of intestinal inflammation, was not available and could not be incorporated into the analysis. This limits the ability to position cytokine measurements within established clinical monitoring frameworks. Integration with established biomarkers such as fecal calprotectin would be essential to better define the incremental clinical value of circulating cytokines. Histologic activity was defined using the Geboes score, which is primarily validated in ulcerative colitis. Its application across both IBD subtypes represents a methodological compromise and may not fully capture disease-specific histologic features, particularly in Crohn’s disease. Finally, circulating cytokine measurements may not fully reflect mucosal immune processes, as local cytokine production within the intestinal microenvironment may differ from systemic levels [47,48,49,50,51,52,53]. Accordingly, systemic cytokine measurements should be interpreted as indirect markers of mucosal immune activity rather than direct surrogates. Despite these limitations, the study has several strengths, including prospective design, consecutive patient inclusion, blinded histologic assessment, and standardized cytokine quantification. The use of prespecified analytical strategies and internal validation further supports the internal consistency of the findings.

Artificial intelligence (AI) is increasingly emerging as a promising tool in the management of inflammatory bowel disease (IBD), with potential applications spanning diagnosis, endoscopic assessment, prognostication, and treatment optimization. Recent advances in machine learning and deep learning have demonstrated the ability to enhance detection of mucosal inflammation, standardize endoscopic scoring, and support risk stratification and personalized therapeutic decision-making. In this context, the integration of AI-driven approaches with systemic biomarker profiling, such as circulating cytokines, may further refine disease monitoring and enable more precise identification of patients with subclinical inflammation or discordant disease states. Although these approaches remain largely investigational, they highlight a potential future direction toward more individualized and data-driven management strategies in IBD [54].

In conclusion, histologic–endoscopic discordance in IBD is associated with a distinct systemic cytokine profile characterized by reduced IL-10 and increased IL-23 levels. These findings suggest that endoscopic remission may not fully capture underlying immune activity and support the concept of integrating systemic immune markers into disease assessment frameworks. Further validation in larger, longitudinal cohorts is required to determine the clinical utility of these observations.

## Figures and Tables

**Table 1:** Baseline demographic, clinical, and laboratory characteristics according to endoscopic–histologic phenotype.

Variable	Concordant Remission (*n* = 22)	Discordant Disease (*n* = 14)	Concordant Active Disease (*n* = 21)	*p* Value
Demographic and clinical characteristics				
Age, years, median (IQR)	49.0 (39.8–55.8)	46.0 (40.3–55.8)	56.0 (32.0–64.0)	0.481
Male sex, *n* (%)	16 (72.7%; 49.8–89.3)	5 (35.7%; 12.8–64.9)	13 (61.9%; 38.4–81.9)	0.085
Disease duration, years, median (IQR)	5.0 (2.3–6.8)	6.5 (2.5–8.8)	4.0 (2.0–10.0)	0.282
Current smoking, *n* (%)	5 (22.7%; 7.8–45.4)	1 (7.1%; 0.2–33.9)	4 (19.0%; 5.4–41.9)	0.475
Laboratory parameters				
C-reactive protein, mg/L, median (IQR)	0.10 (0.10–1.18)	0.50 (0.10–1.95)	6.50 (1.40–10.80)	0.002
Interleukin-10, pg/mL, median (IQR)	4.75 (4.05–5.40)	3.30 (2.80–3.65)	2.30 (1.40–2.60)	<0.001
Interleukin-23, pg/mL, median (IQR)	1.70 (0.30–3.00)	7.25 (5.05–14.83)	10.40 (5.70–13.90)	<0.001
Hemoglobin, g/dL, median (IQR)	13.75 (12.90–14.90)	13.60 (12.00–14.70)	13.20 (12.00–14.70)	0.552
White blood cells, ×10^9^/L, median (IQR)	7.06 (6.24–7.72)	6.13 (5.34–7.54)	7.69 (5.38–9.20)	0.517
Disease characteristics				
Ulcerative colitis, *n* (%)	18 (81.8%; 59.7–94.8)	13 (92.9%; 66.1–99.8)	12 (57.1%; 34.0–78.2)	0.037
Crohn’s disease, *n* (%)	4 (18.2%; 5.2–40.3)	1 (7.1%; 0.2–33.9)	9 (42.9%; 21.8–66.0)	0.037

**Footnote**: Values are presented as median (interquartile range, IQR) or number (%; 95% confidence interval, CI). Continuous variables were compared using the Kruskal–Wallis test. Categorical variables were compared using the χ^2^ test or Fisher’s exact test. CRP denotes C-reactive protein; IL, interleukin; WBC, white blood cells. Montreal classification details are provided in Supplementary Table S1.

**Table 2:** Association Between Endoscopic and Histologic Status (*n* = 59).

	Histologic Activity	Histologic Healing	Total
Endoscopic Activity	21 (91.3%; 72.0–98.9)	2 (8.7%; 1.1–28.0)	23
Endoscopic Remission	14 (38.9%; 23.1–56.5)	22 (61.1%; 43.5–76.9)	36
Total	35	24	59

Pearson χ^2^ test, *p* < 0.001. **Footnote**: Values are presented as number (%), with row percentages. The association between endoscopic and histologic status was assessed using the Pearson χ^2^ test (two-sided).

**Table 3:** Association Between Circulating Cytokines and Discordant Disease (Endoscopic Remission Subgroup, *n* = 36).

Variable	Odds Ratio (95% CI)	*p* Value
Primary multivariable model		
z-standardized log(IL-10)	0.0014 (0.000003–0.576)	0.032
z-standardized log(IL-23)	16.94 (1.90–151.32)	0.011
Sensitivity model		
z-standardized log(IL-10)	0.0052 (0.00005–0.567)	0.028
z-standardized log(IL-23)	11.65 (1.56–86.87)	0.017
C-reactive protein	1.02 (0.73–1.43)	0.885
Male sex	1.88 (0.09–40.71)	0.687

**Footnote**: Odds ratios (OR) with 95% confidence intervals (CI) were estimated using Firth penalized logistic regression. Analyses were restricted to patients in endoscopic remission (*n* = 36), with discordant disease defined as endoscopic remission with persistent histologic activity. The primary multivariable model included z-standardized log-transformed interleukin-10 (IL-10) and interleukin-23 (IL-23) concentrations. The sensitivity model additionally adjusted for C-reactive protein and sex.

**Table 4:** Diagnostic Performance of IL-10 Detectability for Histologic Activity (Endoscopic Remission Subgroup).

Metric	Value (95% CI)
Sensitivity	92.9% (66.1–99.8)
Specificity	77.3% (54.6–92.2)
Positive predictive value	72.2% (46.5–90.3)
Negative predictive value	94.4% (72.7–99.9)

Footnote: Values are presented as percentage (95% confidence interval, CI). Diagnostic performance metrics were calculated for IL-10 detectability in identifying histologic activity among patients in endoscopic remission.

**Table 5:** Exploratory Discriminatory Performance of Models for Discordant Disease.

Model	Variables Included	Apparent AUC	Optimism-Corrected AUC
Base model	CRP	0.578	0.581
Reduced model	IL-10 + CRP	0.925	0.908
Multivariable model	IL-10 + IL-23 + CRP	0.994	0.979

**Footnote**: Area under the receiver operating characteristic curve (AUC) was calculated to assess model discrimination. Internal validation was performed using bootstrap resampling. Calibration was assessed by comparing predicted and observed probabilities. All analyses were restricted to patients in endoscopic remission (*n* = 36). Estimates are exploratory and were not externally validated.

## Data Availability

The datasets generated during the current study are available from the corresponding author on reasonable request. Data are not publicly available due to institutional and ethical restrictions.

## Supplementary Materials

The following supporting information can be downloaded at: https://www.mdpi.com/article/10.3390/jcm15093319/s1, Table S1: Montreal classification according to endoscopic–histologic phenotype.

## Abbreviations

The following abbreviations are used in this manuscript:

IBD	Inflammatory bowel disease
UC	Ulcerative colitis
CD	Crohn’s disease
IL-10	Interleukin-10
IL-23	Interleukin-23
CRP	C-reactive protein
SES-CD	Simple Endoscopic Score for Crohn’s Disease
ROC	Receiver operating characteristic
AUC	Area under the curve
LOD	Limit of detection
LLOQ	Lower limit of quantification
OR	Odds ratio
