# Supplementary material for: Systemic Immune Signatures of Endoscopic–Histologic Discordance in Inflammatory Bowel Disease: A Pilot Study
Source: J Clin Med. 2026 Apr 27;15(9):3319. doi: 10.3390/jcm15093319 (PMC13163518; doi:10.3390/jcm15093319)
Supplement: Supplementary file 1 [file jcm-15-03319-s001.zip › jcm-4248011-supplementary.pdf]

**Table S1. Montreal classification according to endoscopic–histologic phenotype**

| Variable | Concordant Remission (*n*=22) | Discordant Disease (*n*=14) | Concordant Active Disease (*n*=21) | P value |
|---|---|---|---|---|
| **Ulcerative colitis – Extent (Montreal)** | | | | 0.651 |
| **E1** | 3 (13.6) | 1 (7.1) | 0 (0.0) | — |
| **E2** | 1 (4.5) | 1 (7.1) | 1 (4.8) | — |
| **E3** | 14 (63.6) | 11 (78.6) | 11 (52.4) | — |
| **Crohn's disease – Location (Montreal)** | | | | 0.223 |
| **L1** | 1 (4.5) | 0 (0.0) | 6 (28.6) | — |
| **L2** | 0 (0.0) | 0 (0.0) | 0 (0.0) | — |
| **L3** | 3 (13.6) | 1 (7.1) | 3 (14.3) | — |

**Footnote:** Values are presented as number (%). Percentages are calculated within each phenotype group.
